# Phosphoproteomics-Mediated Identification of Fer Kinase as a Target of Mutant Shp2 in Noonan and LEOPARD Syndrome

**DOI:** 10.1371/journal.pone.0106682

**Published:** 2014-09-03

**Authors:** Jeroen Paardekooper Overman, Christian Preisinger, Karin Prummel, Monica Bonetti, Piero Giansanti, Albert Heck, Jeroen den Hertog

**Affiliations:** 1 Hubrecht Institute-Koninklijke Nederlandse Akademie van Wetenschappen and University Medical Center Utrecht, Utrecht, The Netherlands; 2 Biomolecular Mass Spectrometry and Proteomics, Bijvoet Center for Biomolecular Research and Utrecht Institute for Pharmaceutical Research, Utrecht University, Utrecht, The Netherlands; 3 Netherlands Proteomics Centre, Utrecht, The Netherlands; 4 Proteomics Facility, Interdisciplinary Centre for Clinical Research Aachen, Aachen University, Aachen, Germany; 5 Centre for Biomedical Genetics, Utrecht, The Netherlands; 6 Institute Biology Leiden, Leiden, The Netherlands; University of Sheffield, United Kingdom

## Abstract

Noonan syndrome (NS) and LEOPARD syndrome (LS) cause congenital afflictions such as short stature, hypertelorism and heart defects. More than 50% of NS and almost all of LS cases are caused by activating and inactivating mutations of the phosphatase Shp2, respectively. How these biochemically opposing mutations lead to similar clinical outcomes is not clear. Using zebrafish models of NS and LS and mass spectrometry-based phosphotyrosine proteomics, we identified a down-regulated peptide of Fer kinase in both NS and LS. Further investigation showed a role for Fer during development, where morpholino-based knockdown caused craniofacial defects, heart edema and short stature. During gastrulation, loss of Fer caused convergence and extension defects without affecting cell fate. Moreover, Fer knockdown cooperated with NS and LS, but not wild type Shp2 to induce developmental defects, suggesting a role for Fer in the pathogenesis of both NS and LS.

## Introduction

Noonan syndrome (NS) (OMIM 163950) is a congenital disorder that manifests itself in heart defects, short stature, webbed neck, hypertelorism and an increase in the occurrence of juvenile myelomonocytic leukemia (JMML) and other malignancies. The most common causes for NS are mutations in *PTPN11* encoding for Src-homology domain 2 (SH2) containing phosphatase 2 (Shp2) [1] A similar syndrome is also caused by mutations in *PTPN11* and patients display similar symptoms as NS. An acronym of the symptoms, Lentigines, Electrocardiographic conduction anomalies, Ocular hypertelorism, Pulmonary stenosis, Abnormal genitalia, Retarded growth and Deafness gave this syndrome its name, LEOPARD syndrome (LS)(OMIM 151100) [1]. Both NS and LS are part of a group of congenital syndromes caused by mutations in the RAS mitogen activated protein kinase (MAPK) pathway called RASopathies. Despite the similarities in the clinical manifestations of NS and LS, NS mutations lead to an ‘active’ form of the Shp2 phosphatase, while LS is thought to result from ‘inactivating’ mutations [2], [3]. However, a gain-of-function for LS has also been described in *Drosophila* [4].

The phosphatase Shp2 consists of two N-terminal SH2 domains that are able to bind to tyrosine phosphorylated targets of Shp2, a protein tyrosine phosphatase (PTP) domain, and a C-terminal tail with a proline rich domain and two tyrosines that are able to bind Grb2 when phosphorylated. Under basal conditions, the N-SH2 domain blocks access of the PTP domain to its substrates, resulting in an inactive conformation of Shp2 [5]. In NS, mutations cause a disruption of the interaction between the N-SH2 domain and the PTP domain, resulting in a hyperactive form of Shp2 [1], [2]. In LS however, mutations are mostly present in the PTP domain at the interface of the PTP and SH2 domain, resulting in a loss of catalytic activity. How both activating and inactivating mutations of Shp2 lead to similar developmental defects is largely unknown.

Homozygous mice defective for Shp2 die *in utero* and have gastrulation defects, showing malformations of the notochord and posterior truncations [6]. Embryos lacking Shp2 also develop failure of neural tube closure resulting in *spina bifida* and secondary neural tubes resulting from gastrulation defects [7]. Shp2 is essential for limb formation since chimeric mice with defective Shp2 expressed in the mesenchyme of the progress zone showed limb bud defects [8]. More recent results have shown cell migration defects during gastrulation in NS and LS Shp2 expressing zebrafish embryos as well [9]. These convergence and extension (C&E) cell movements mediate the anterior-posterior lengthening and lateral narrowing of the developing embryo. C&E cell movements are under strict spatiotemporal control of various signaling pathways [10]. Being a protein proximal to many RTKs, Shp2 acts upstream of multiple signaling pathways [2].

As NS and LS Shp2 are thought to act biochemically opposite, yet give rise to similar clinical symptoms, we sought to identify potential common targets of NS and LS Shp2 in a zebrafish model. Pinpointing disease associated Shp2 signaling shared by NS and LS, may contribute to the understanding of the underlying mechanisms of NS and LS pathogenesis and the development of therapeutic strategies for both NS and LS. Using a comparative phosphoproteomics approach, phosphotyrosine (pTyr)-containing peptides of NS- and LS-Shp2 expressing zebrafish embryos were isolated, identified and compared to peptides of WT embryos. A peptide corresponding to the autophosphorylation site of Fer kinase was identified as one of the most down-regulated peptides in developing NS and LS zebrafish. Further investigation revealed a role for Fer in C&E cell movements during gastrulation. Downregulation of Fer cooperated with NS and LS to induce developmental defects, suggesting a genetic interaction between Fer and the NS and LS variants of Shp2.

## Materials and Methods

### Ethics statement

Only embryos up to 4 days post fertilization (dpf) were used for these experiments, which do not require approval of the animal experiments committee according to national and European law.

### Zebrafish maintenance and *in situ* hybridization

Wild type and *Tg(−4.9sox10:EGFP)^ba2^* zebrafish were kept under standard conditions. Embryos were staged as described by Westerfield [11]. *In situ* hybridizations were done as described before [9], using probes specific for *bmp2b*, *chd*, *cyc, gsc*, *krox20*, *myod, ntl*, *pax2* and *six3*. For the *fer* probe, Fer-pBSK- was digested with NotI for the antisense probe and with ApaI for the sense probe, respectively. T7 and T3 RNA polymerase were used to generate digoxigenin labeled RNA (Roche). Embryos were fixed at the indicated stages with 4% paraformaldehyde (PFA). Embryos were treated from 24 hpf onwards with 0.2 mM 1-phenyl 2-thiourea (Sigma) in E3-medium to block pigmentation.

### Constructs

*fer* was amplified from cDNA of 1 dpf WT embryos and cloned into EcoRI/XhoI sites of pCS2+ and pBSK- and verified by sequencing using: Fer_out_Fw: 5′-GAGAGAGACTGCGTGCCTTG-3′, Fer_out_Rv: 5′-ATGAGATTCTGAGGGCGAAA-3′ and Fer_EcoRI_Fw: 5′-GGCGAATTCATGGGGTTCGGCCGGGAC-3′, Fer_XhoI_Rv: 5′-CGGCTCGAGTTAGGGATCACCTGGATG-3′. The mutation Y716F was introduced into *fer* by site-directed mutagenesis using Fer_Y716F_Fw: 5′-TCCTGATGAGGAGAAGATGCCGTCGTC-3′ and Fer_Y716F_Rv: 5′-GACGACGGCATCTTCTCCTCATCAGGA-3′.

### Morpholinos, RNAs and injections

Fer splicing morpholinos (MOs) were designed for several intron-exon boundaries and ordered from GeneTools (Philomath, OR, USA). The p53 MO (5′-GCGCCATTGCTTTGCAAGAATTG-3′) was described before and was used as recommended by the manufacturer [12]. Synthetic mRNA was synthesized using mMessage mMachine kit (Ambion). pCS2+ plasmid was digested with NotI and RNA was synthesized with SP6 RNA polymerase. The NS and LS constructs were previously described [9]. MOs and mRNA was injected at one cell stage and the working concentration was titrated for each MO and mRNA. Fer MO i5e6 (5′-GGCTTCTGGTGATCTGTTGAAATA-3′) was used at 1.0 ng/µl (high concentration) or 0.5 ng/µl (low concentration). Fer MO e9i9 (5′-GGGATTACACTTACTGACGAAGAGC-3′) was used at 2.5 ng/µl (high concentration) or 1.0 ng/µl (low concentration). For co-injection, suboptimal concentrations of both MOs were combined.

### Alcian blue staining

Embryos were anesthetized with tricaine methane sulfonate (Sigma) and fixed in 4% PFA at 4 dpf. After several hours of fixing, embryos were washed for 10 min in 50% ethanol. Embryos were stained overnight at 4 degrees in staining solution (0.04% Alcian blue (Sigma), 70% ethanol and 50 mM magnesiumchloride) and washed in 0.2% Triton X-100 (Sigma). Afterwards, embryos were bleached to remove pigmentation (8.5% hydrogen peroxide, 5% formamide, 0.5×SSC) and washed with 0.2% Triton X-100. Images were quantified by using ImageJ software.

### RNA isolation

Total RNA of 3 dpf Nacre MO and Fer MO injected embryos was isolated. Approximately 30 embryos were dissolved in 1.0 ml TriZol (Invitrogen). The homogenate was centrifuged (12.000 g, 4 degrees, 10 min). 0.2 ml Chloroform was added to the supernatant, vortexed for 15 sec, incubated for 3 min at room temperature (RT) and afterwards centrifuged (12.000 g, 4 degrees, 15 min). 0.5 ml isopropanol was added to the upper phase, incubated for 10 min at RT and centrifuged (12.000 g, 4 degrees, 15 min). The pellet was washed with 75% ethanol and after vortexing centrifuged (10.000 g, 4 degrees, 5 min). The pellet was dried for 5 min and dissolved in 100 µl deionized water. Subsequently, the solution was incubated for 10 min at 37°C, precipitated in 250 µl 100% ethanol and 10 µl 3 M sodium acetate and stored at −80°C.

### RT-PCR

For the reverse transcription reaction (RT-PCR) with M-MLV-RT (Promega) 1 µg of total RNA was used. PCR of cDNA was performed using standard protocol of GoTaq (Promega) using Fer_Fw_intron_5: 5′-TTTCTGTCCCCATTGCAAAG-3′, Fer_Rv_exon_7: 5′-GATTCTGCTGGGTTATTAGC-3′, Fer_Fw_exon_6: 5′-GAACCTAATGTAGAATTTGATGC-3′, Fer_Fw_exon_8: 5′-CGGAGGCCAAACTCATGGCACA-3′ and Fer_Rv_exon_10: 5′-CGCACCAGAAAGTCTCCCTG-3′. *Gadph* was used as a loading control for the RT-PCR reaction.

### Mass spectrometry

#### Digest Preparation

Embryos were injected at the 1-cell stage with 150 pg wild type Shp2, 150 pg Shp2-D61G or 50 pg Shp2-A462T RNA, respectively. Shp2 RNA injected embryos were co-injected with enhanced green fluorescent protein (GFP) RNA used for screening of injection efficiency. Embryos expressing GFP were selected, manually dechorionated and collected at 26 hpf to 28 hpf. Embryos were then deyolked and washed using deyolking buffer (1/2 Ginzburg Fish Ringer) without calcium (2×10 µl/embryo) snap frozen and stored at −80°C for further usage [11], [13]. Embryo pellets corresponding to a total of approximately 2000 embryos per condition were resuspended and lysed in 8 M Urea, 50 mM ammoniumbicarbonate, 1 PhosSTOP tablet per 10 ml plus 1 mM vanadate and ethylenediaminetetraacetic acid-free protease inhibitor cocktail (Sigma). Embryo lysates were sonicated for 4×30 seconds and then centrifuged at 14 500 rpm for 30 minutes at 4°C.

Lysate supernatants equivalent to 3 to 4 mg total protein per condition were generated in principle as described previously [14], [15]. Briefly, the lysates were reduced with dithiothreitol (10 mM) for 1 hour at 56°C and alkylated with iodoacetamide (55 mM) at room temperature, in the dark for 45 minutes and digested for four hours with the protease Lys-C (1∶100 enzyme/substrate) at 37°C. Samples were then diluted 4 fold with 50 mM Ammoniumbicarbonate to 2 M Urea and trypsin (1∶100) was added and digestion was performed overnight at 37°C. The peptide mixtures were acidified to pH 3 by adding acetic acid to a final concentration of 2.5%. The individual peptide solutions were then desalted, dimethyl labelled on-column as described previously [16]. Wild type Shp2 injected embryos were labeled “light” whereas D61G (Noonan) Shp2 and A462T (LEOPARD) Shp2 injected embryos were labeled “intermediate” and “heavy”, respectively.

#### Immunoprecipitation (IP)

IP was performed as described [17]. In principle, labeled peptide solutions were mixed in equal concentrations, vacuum dried and resuspended in IP Buffer (50 mM Tris, pH 7.4, 150 mM sodium cloride, 1% n-octyl-β-D-glucopyranoside and a Complete Mini protease inhibitor tablet per 10 ml IP buffer (Roche Diagnostics)). PY99-agarose beads (Santa Cruz Biotechnology) were washed five times with IP buffer prior to IP. The labeled peptide mixture was mixed with the PY99-agarose beads, and incubation was performed overnight at 4°C under constant rotation. After peptide immunoprecipitation, the beads were washed three times with IP buffer and two times with ultrapure water. Peptides were eluted by adding two times 50 µl of 0.15% trifluoroacetic acid for 20 min at room temperature. Eluted peptides were then desalted and concentrated on C18 tips and resuspended in 10% formic acid prior to MS analysis.

#### On-line Nanoflow Liquid Chromatography

As described in [17]: Nanoflow LC-MS/MS was performed on an Linear Trap Quadrupole-Orbitrap XL mass spectrometer (Thermo Electron, Bremen, Germany) coupled to an Agilent 1100 HPLC system (Agilent Technologies, Waldbronn, Germany). Dried peptides were trapped at 5 µl/min in 100% solvent A (0.1 M acetic acid in water). Subsequently, peptides were transferred to an analytical column (ReproSil-Pur C_18_- AQ, 3 µm (Dr. Maisch GmbH, Ammerbuch, Germany); 40 cm * 50 µm inner diameter, packed in house) at ∼100 nl/min and eluted using a 3-h gradient from 0 to 40% solvent B (0.1 M acetic acid in 8∶2 (v/v) acetonitrile/water). The eluent was sprayed via distal coated emitter tips (New Objective) butt-connected to the analytical column. The LTQ Orbitrap XL was operated in data-dependent mode, automatically switching between MS and MS/MS. Full-scan MS spectra (from m/z 350 to 1500) were acquired in the Orbitrap with a resolution of 60,000 at m/z 400 after accumulation to target value of 500,000. The three most intense ions at a threshold above 500 were selected for collision-induced fragmentation in the linear ion trap at a normalized collision energy of 35% after accumulation to a target value of 10,000.

#### Data Analysis

All MS_2_ spectra were processed and quantified with Proteome Discoverer (version 1.3.0.399). Runs were searched against a concatenated forward-decoy version of the Zebrafish Uniprot database (version 20130916, 81920 sequences) with Mascot (version 2.3.02, Matrix Science). The database search was performed with the following parameters: a mass tolerance of ±50 ppm for precursor masses and ±0.6 Da for CID fragment ions, allowing two missed cleavages, cysteine carbamidomethylation as fixed modification. Light, intermediate and heavy dimethylation of peptide N-termini and lysine residues; methionine oxidation; phosphorylation on serine, threonine and tyrosine were set as variable modifications. The enzyme was specified as trypsin. The phosphorylation site localization of the identified phosphopeptides was performed by the phosphoRS algorithm 3.1 implemented in Proteome Discoverer using a 75% cut-off. For phosphopeptides that did not have a phosphorylation site with a pRS score above 75%, the site was counted as “ambiguous”. The dimethyl-based quantitation method was chosen in Proteome Discoverer, with mass precision requirement of 2 ppm for consecutive precursor mass measurements. A 0.5 min retention time tolerance was applied for isotope pattern multiplets and allowed spectra with maximum 1 missing channels to be quantified. Mascot results were further filtered with the following criteria: (i) mass deviations of ±10 ppm; (ii) Mascot ion score of at least 20; (iii) a minimum of 6 amino-acid residues per peptide; and (iv) position rank 1, which results in a peptide FDR<1%. Phosphopeptides that were found to be differentially phosphorylated were manually validated.

#### Quantification

Quantified phosphopeptides were normalized against the median of all non-phosphopeptides. For peptides with phosphorylation sites with a pRS score above 75%, only these phosphopeptides were used for quantification. For peptides with only pRS scores below 75% all phosphopeptides were used for quantification, but the phosphorylation site was counted as “ambiguous”. Log2 ratios of heavy/light and medium/light of more than 1 or less than −1 were accepted as significant changes in phosphorylation levels.

#### Annotation

Phosphorylation sites of each peptide were compared to the total protein sequence of the corresponding zebrafish protein to identify the phosphorylation site. These sites were then compared to human protein in Phosphosite.org. For ambiguous phosphorylation sites (pRS<75) the sequence was compared to Phosphosite.org to identify the most commonly identified site. Non-annotated peptides were BLASTed against the zebrafish proteome to identify the protein.

## Results

### Mass spectrometric analysis identifies Fer kinase as a hypotyrosyl-phosphorylated protein in NS and LS zebrafish embryos

**Figure 1:**
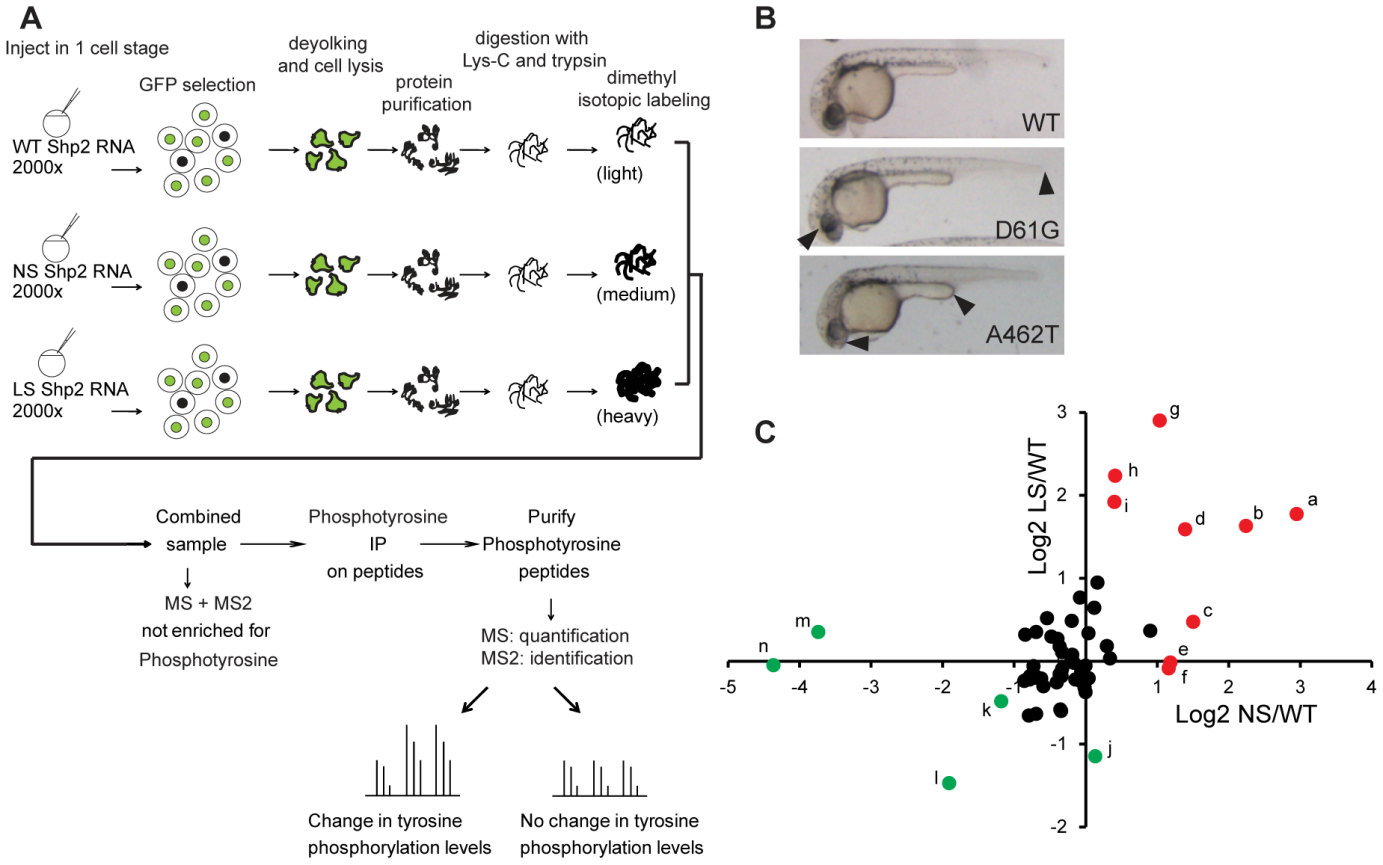
Comparative pTyr mass spectrometry on 1 day old zebrafish embryos expressing wild type, NS (D61G) or LS (A462T) Shp2. A. Work flow depicting the mass spectrometry approach. Approximately 2000 zebrafish embryos per condition were injected at the 1-cell stage and sorted for GFP expression. Embryos were lysed, trypsinised and labeled using the dimethyl labeling method. WT, NS and LS samples were combined and immunoprecipitated using pTyr specific antibodies. Immunoprecipitate was subjected to MS and peptides were identified and quantified based on MS2 and MS1 spectra, respectively. B. 1 dpf zebrafish embryos expressing WT, D61G and A462T Shp2. Body axis length, craniofacial defects and heart edema in D61G and A462T Shp2 expressing zebrafish are indicated with arrowheads. C. Normalized plot of quantified phosphopeptides Log2 ratios. Peptide ratios with Log2 ratios >−1 and <1 are indicated in black. Peptides with more that 1× Log2 difference are annotated with a-n (see Table 1 for reference). Peptides changed with Log2 ratios <−1 in either NS or LS are indicated in green and Log2 ratios >1 in either NS or LS are indicated in red. See text for further details.

To identify novel downstream targets of disease associated Shp2 that are affected similarly in NS and LS, we performed a mass spectrometric analysis on zebrafish embryos as outlined in Figure 1A. In short, zebrafish embryos were injected with synthetic mRNA encoding wild type Shp2, NS-Shp2 (D61G) or LS-Shp2 (A462T) which induced developmental defects at 24 hpf [9] (Figure 1B). Embryos were co-injected with mRNA encoding GFP, which facilitated selection of embryos that were injected properly. Approximately 2000 embryos per condition were lysed and combined prior to digestion with LysC and Trypsin. Zebrafish lysates were isotopically labeled with normal formaldehyle (CH_2_O, light) for the WT embryos, deuteroformaldehyde (CD_2_O, medium) for the NS embryos and 13C deuteroformaldehyde (^13^CD_2_O, heavy), for LS embryos. Labeled peptides were then combined and the mixture was processed further. The labeled peptide mixtures were enriched for pTyr by immunoprecipitation, using PY99 agarose beads [17], [18] (Figure 1A).

LC-MS was performed and MS1 and MS2 spectra were obtained. Ratios of light/medium and light/heavy peptides were determined with Proteome Discoverer 1.3.0.399 using the MS^1^ spectra and normalized to the total ratio of all (non-phosphorylated) peptides as input. The input corresponding to the LEOPARD Shp2 expressing embryos (heavy labeled) was 2.35 fold higher than wild type (light labeled), whereas Noonan (medium labeled), was 1.38 fold higher than wild type. Whereas many peptides were identified, only a small number of phosphopeptides were identified and of suitable quality for quantification (see Table S1). In total, we identified and quantified 69 phosphorylation sites, of which 44 were pTyr sites with a pRS score of >75%. The other phosphorylation sites were predicted to be on serine or threonine, or the pTyr site could not be localized. The low abundance of tyrosine phosphorylation is likely the cause of the low number of identified and quantified pTyr peptides. In addition, many peptides that were identified corresponded to highly abundant proteins like actin, keratin and vitellogenin, a zebrafish yolk protein. These abundant proteins may have interfered with binding of the antibody to peptides of interest, leading to reduced specificity/efficiency for the enrichment of pTyr containing peptides.

The normalized Log2 ratios of the quantified phosphopeptides indicate that the majority of phosphopeptides was unaltered upon expression of NS or LS Shp2 compared to WT Shp2 (Table 1, Figure 1C). For clarity, the human phosphorylation sites are used in the text. A minor fraction of phosphopeptides was up- or down-regulated in NS and LS Shp2 expressing zebrafish embryos, compared to WT Shp2 expressing embryos. This fraction of peptides may represent downstream factors that contribute to the etiology of both NS and LS. As an internal control for the expression of NS and LS Shp2 compared to wild type, we confirmed similar levels of Shp2 Y63 phosphopeptide in the WT compared to LS. Indeed, the ‘WT’ Shp2 pY63 peptide was not observed in the medium labeled (NS) sample, as this peptide has a D61G substitution and was thus not identified (See Table 1, Figure 1B, peptides “m” and “n”).

**Table 1:** Comparative mass-spectrometry of pTyr immunoprecipitated zebrafish lysates.

Protein name	accession number	Identified peptide sequence	Site in zebrafish	Site in human	NS/WT Log2	LS/WT Log2	
Mpzl1	E7F7T2	CSSPSAPVQGPVI(pY)AQLDHSGSK	Y236	Y241	2,95	1,77	a
Mpzl1	E7F7T2	MEPVV(pY)ADIR	Y258	Y263	2,24	1,63	b
Histone 2B 1/2	Q5BJA5	ESYAIYV(pY)K	Y43	Y43	1,50	0,47	c
Irs1b*	XP_001920152.3*	TGSD(pY)MNMSPISAR	Y689	Y989	1,39	1,59	d
zgc:171775	F1QHF2	HTETEM(pT)GYVVTR	T181	T183	1,18	−0,02	e
zgc:171775	F1QHF2	HTETEMTG(pY)VVTR	Y183	Y185	1,16	−0,09	f
Ephb3*	XP_005174117.1*	FLEDDPTDPTYTSSLGGK	Y737**	Y792**	1,04	2,90	g
wu:fc15a01	E7F486	GPLDGSL(pY)AQVK	Y478	Y483	0,90	0,37	
Eif3c	NP_998628.1*	QQALLL(pS)DDEEDTK	S39	S39	0,41	2,24	h
Cdk15; Cdk16*	NP_001035398.1; XP_003199208.1*	LGEGTYATVYK	Y99; Y148**	Y114;Y176**	0,40	1,92	i
Prpf4ba	B0V1K6	LCDFGSASHVADNDITPYLV(pS)R	S855	S852	0,34	0,03	
Prpf4ba	B0V1K6	LCDFGSASHVADNDITP(pY)LVSR	Y852	Y849	0,30	0,18	
Rplp; Rplp2l	Q6P5K5	KEESEE(pS)DDDMGFGLFD	S80	S104	0,17	0,95	
Ror2	E7FCE4	WMSPEAIL(pY)GK	Y668	Y666	0,13	−1,15	j
Fynrk	E7F1M5	LDNGGY(pY)LSTAR	Y181	Y214	0,12	0,64	
Histone 2B 1/2	Q5BJA5	ESYAI(pY)VYK	Y39, Y41	Y41	0,04	−0,21	
Pard3	A2BEM7	TLSP(pS)PDDHER	S705	S695	0,04	0,34	
Mapk14a	Q9DGE2	HTDDEMTG(pY)VATR	Y183	Y182	0,00	−0,06	
Flo11*	XP_005172506.1*	TEEDHV(pY)SFPNK	Y139	Y118	0,00	−0,37	
Hmga1*	NP_998333.1*	KHPQQEASG(pS)PTPK	S36	S49	−0,01	−0,32	
Mapk14a	Q9DGE2	HTDDEM(pT)GYVATR	T181	T180	−0,03	−0,07	
Fynrk	E7F1M5	ELVEHY(pS)K	S199	Y193	−0,06	−0,13	
Dyrk2	Q5RHV3	VYT(pY)IQSR	Y379	Y382	−0,07	−0,24	
Krt8	Q6NWF6	NFSSLSYSGPSMSR	Y29**	Y25**	−0,08	0,77	
Mapk12b	Q5RHW0	QADSEMTG(pY)VVTR	Y183	Y185	−0,14	−0,22	
Peak1	E7F7V3	AT(pY)TNLGQSR	Y1096	Y1107	−0,17	−0,02	
Dyrk1b; Dyrk1ab	D1L3Y5;A9QVW5	IYQ(pY)IQSR	Y326; Y273	Y321	−0,19	0,00	
Fynrk	E7F1M5	LDNGG(pY)YLSTAR	Y180	Y213	−0,19	0,08	
Ybx1	A1A605	REGAE(pS)APEGEMQQQQR	S159	S176	−0,20	0,48	
Hipk2	Q1MTA8	AVCST(pY)LQSR	Y375	Y352	−0,33	−0,09	
Prkcda	Q7ZUC5	RPDNNTQDVGV(pY)QDFNK	Y314	Y313	−0,33	0,11	
Mapk14b	Q9DGE1	LTDDEM(pT)GYVATR	T181	T180	−0,34	−0,18	
Mapk11	Q6IQ84	QTDDEMTG(pY)VATR	Y181	Y182	−0,34	−0,13	
Hipk2; Hipk3	Q1LUZ7;Q1MTA8	AVCSTYLQ(pS)R	S366	S355	−0,34	−0,14	
Mapk12	O42376	QTDSEMTG(pY)VVTR	Y183	Y185	−0,34	−0,60	
Mapk12	O42376	QTDSEM(pT)GYVVTR	T181	T183	−0,35	−0,58	
Afap1l2	E7FH23	SSNAGGDEE(pY)I(pY)MNK	Y55, Y57	Y54, Y56	−0,36	0,18	
Mapk14b	Q9DGE1	L(pT)DDEMTGYVATR	T176	T175	−0,36	−0,16	
Eppk1	I3ISA6	AVTGYTDP(pY)TGK	Y1642	Y1656	−0,39	0,27	
Mapk14b	Q9DGE1	LTDDEMTG(pY)VATR	Y183	Y182	−0,40	−0,26	
Cdk1; Cdk2	Q7T3L7;Q7ZWB1	IGEGTYGVV(pY)K	Y19; Y19	Y19; Y19	−0,48	0,29	
Ctnnd	XP_005160310.1*	YRPVDG(pY)R	Y215	Y248	−0,54	0,52	
Hck	E7F0I7	IIEDNE(pY)TAR	Y384	Y419	−0,59	−0,30	
Cdk1; Cdk2	Q7T3L7;Q7ZWB1	IGEGT(pY)GVVYK	Y15	Y15	−0,62	−0,21	
Ptk2.1	F1QT14	YMEDS(pS)(pY)YK	S579, Y580	S576, Y577	−0,69	−0,64	
Keratin 4	F1QK60	GYTSQ(pS)AYAVPAGSTR	S22	S51	−0,69	0,35	
Chrnb1*	NP_001240739.1*	VADE(pY)FIR	Y380	Y390	−0,70	−0,18	
Abl1; Abl2	E7FDC6;B0UXN7	LMTGDT(pY)TAHAGAK	Y412; Y407	Y393; Y439	−0,70	−0,20	
Ptk6a	F1Q7D7	ASACEPGSEL(pY)K	Y111	Y13	−0,73	−0,06	
Gsk2aa; Gsk3b	Q9YH61;Q9YH60	GEPNVS(pY)ICSR	Y216	Y216	−0,78	−0,18	
Ptk2.1	F1QT14	YMED(pS)SYYk	S578	S575	−0,78	−0,22	
Ptk6a	F1Q7D7	ESV(pY)SSEDAQIPYK	Y446	Y432	−0,79	−0,66	
Ptk2.1	F1QT14	YMEDSS(pY)YK	Y580	Y577	−0,82	−0,22	
Pfn2	Q802D5	SQGGEPT(pY)NIAVGK	Y99	Y99	−0,85	0,32	
Abl1; Abl2	E7FDC6;B0UXN7	LMTGDTY(pT)AHAGAk	T413, T408	T394; T440	−0,86	−0,24	
Irs1b*	XP_005157831.1*	SSD(pY)MPMSPK	Y584	Y612	−1,18	−0,48	k
Fer	F1QBS0	QEDDGIYSSSGLK	Y716**	Y714**	−1,91	−1,47	l
Ptpn11a	F1QZU5	IQNTGD(pY)YDLYGGEK	Y60	Y62	−3,74	0,35	m
Ptpn11a	F1QZU6	IQNTGDY(pY)DLYGGEK	Y61	Y63	−4,36	−0,05	n

Zebrafish embryos were injected at the 1-cell stage with synthetic mRNA constructs encoding WT Shp2, NS (D61G) Shp2 or LS (A462T) Shp2 and co-injected with mRNA encoding eGFP. Lysates were subjected to mass spectrometry as described in Materials and Methods. Normalized ratios (Log2 scale) based on total levels of non-phosphorylated peptides are given.

*Protein name based on BLAST sequence of peptide. Accession numbers from BLAST hits are used for non-annotated peptides.

**pRS score <75, phosphorylation site could not be determined but the most commonly identified site from Phsophosite.org is used. a-n: indicators for Figure 1C.

Direct targets of Shp2 PTP activity were expected to have decreased pTyr in NS and an increase in LS. Surprisingly, these peptides were not abundant in our results (see Figure 1C upper-left quadrant). However, we identified pTyr peptide Y612 of IRS1B (peptide “k”), a known Shp2 substrate, which was decreased (Log2: −1.18 fold) in NS but unaltered in LS compared to WT. Peptides that were increased in LS included a putative phoshorylation site of cell division kinase 15 and/or 16 (CDK15/CDK16)(peptide “i”)(Log2: 1.92 fold). However, these sites could not be localized with a precision score of >75%. In addition, S39 of eukaryotic translation initiation factor 3 subunit C (EIF3C) (peptide “h”) was increased (Log2: 2.24 fold) in LS.

Alternatively, proteins that are phosphorylated downstream of Shp2 in a phosphatase-dependent manner would be expected in the lower right quadrant with increased phosphorylation in response to NS Shp2 and decreased phosphorylation in response to LS Shp2. We identified phosphopeptides that were increased in NS, including Y43 of Histone 2B (H2B) (peptide “c”) (Log2: 1.50 fold), Y185 (Log2: 1.16 fold) (peptide “f”) and T183 (Log2: 1.18 fold) (peptide “e”) of a MAPK12/MAPK14 protein (zgc:171775). Additionally, we identified a phosphopeptide that was decreased in LS including Y666 of receptor tyrosine kinase-like orphan receptor 2 (ROR2) (peptide “j”) (Log2: −1.15 fold).

Multiple peptides that were affected in a similar way (upregulation or downregulation in both NS and LS) were identified (lower-left and upper-right quadrant). To gain further understanding in how activating and inactivating mutations in NS and LS, respectively lead to similar outcomes, we chose to focus on this group of peptides. Two of the main hyperphosphorylated peptides in both NS and LS corresponded to PZR, an earlier identified target of Shp2 [19], [20]. Peptides corresponding to Y241 (peptide “a”) and Y263 (peptide “b”) were increased (Log2: 2.95 fold and Log2: 2.24 fold, respectively in NS and Log2: 1.77 and Log2: 1.63 fold, respectively in LS). A further analysis of the role of PZR in NS and LS is described elsewhere [21]. In addition, we identified a pTyr peptide corresponding to Y989 in human Insulin receptor substrate 1B (IRS1B) to be highly abundant in NS (Log2: 1.39 fold) and LS (1.59 fold) (peptide “d”). A phosphopeptide corresponding to Y792 of EphB3 (peptide “g”) was also upregulated in NS and LS (Log2: 1.04 fold in NS and Log2: 2.90 in LS).

At the other end of the spectrum, the most decreased phosphopeptide in NS and LS corresponded to Fer kinase (peptide “l”, NS: Log2 −1.91 fold, LS: Log2 −1.47 fold). The MS^2^ spectrum of the Fer phosphopeptide is given in Figure S1. Unfortunately, a reference peptide for total Fer levels was not identified. Thus, the reduced levels of Fer phosphopeptide may represent reduced Fer phosphorylation, reduced total Fer levels or a combination of both. While phosphorylation of serine instead of tyrosine in this peptide could not be excluded, the tyrosine in this peptide is found frequently in other mass spectrometry based experiments (http://www.phosphosite.org). In addition, the peptide was immunoprecipitated with anti-pTyr antibodies, which indicates that this tyrosine is likely phosphorylated. Interestingly, this tyrosine is the autophosphorylation site of Fer [21]. Since Fer had not been studied in zebrafish nor had Fer been implicated in NS and LS, we further investigated the role of Fer in these syndromes.

### Fer kinase expression in zebrafish embryos and Fer MO induced splicing defects

The Fujinami poultry sarcoma (fps)/Feline sarcoma (fes) related kinase (Fer) is a widely expressed, 94 kDa non-transmembrane protein tyrosine kinase (PTK) that is associated with cell migration, tumor growth and was first described in 1986 [22]–[24]. Fer is involved in focal adhesion and cadherin signaling, as well as ERK, Akt and Stat3 signaling [22]. Fer and Shp2 share many signaling pathways and a requirement for both proteins has been described in synapse formation [25]. It is thus likely that signaling between Fer and Shp2 plays an important role in cell-cell adhesion, migration and morphogenesis.

**Figure 2:**
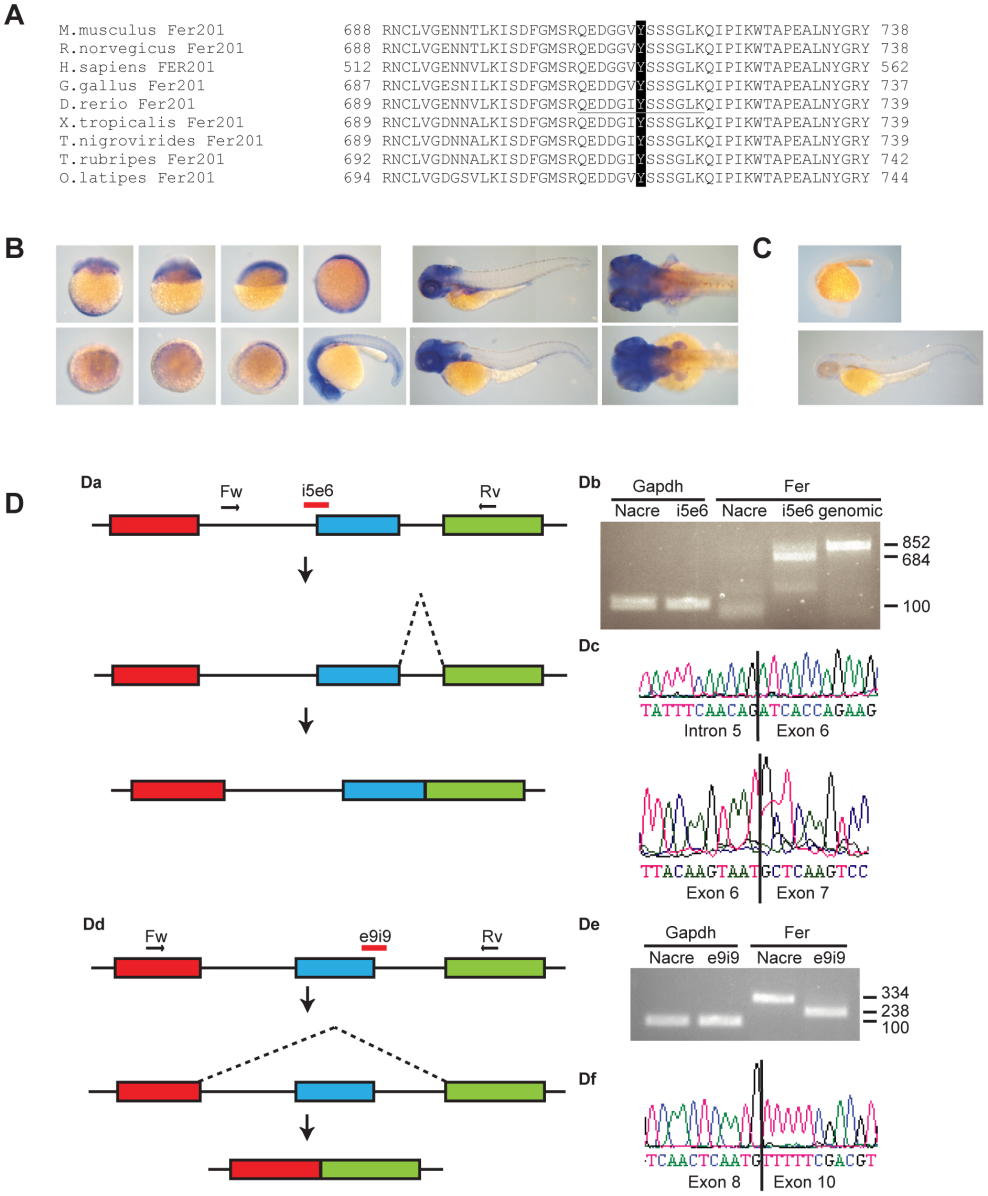
Fer expression in zebrafish embryos and Fer MO induced splicing defects. A. Alignment of zebrafish Fer kinase sequence encompassing the autophosphorylation site (black) that was identified by phosphoproteomics with the mammals *M. musculus*, *R. norvegicus*, *H. sapiens*, the avian *G. gallus*, the amphibian *Xenopus tropicalis*, and the fish *Tetraodon nigrovirides, Takifugu rupripes* and *Oryzias latipes*. B. *fer* expression during the first 24 hpf and at 3 dpf and 4 dpf was observed using *in situ* hybridization with an antisense *fer* probe. C. ISH with negative control sense *fer* probe. D. RT-PCR showing altered splicing of *fer* in MO injected embryos. Da. Model of altered splicing by Fer MO i5e6. Exons 5, 6 and 7 are indicated in red, blue and green, respectively. Primers used for RT-PCR are indicated as arrows. MO is indicated in red. Due to splice blocking, intron 5 is not spliced out of the processed mRNA. Db. RT-PCR showing *gapdh* control in Nacre (control) MO and Fer MO injected embryos. RT-PCR showing *Fer* product in Nacre control MO and Fer i5e6 MO injected embryos, and genomic DNA as a positive control. Dc. Sanger sequencing showing the inclusion of intron 5 and the normal splicing of exon 6 in the RT-PCR product. Dd. Model of altered splicing by Fer MO e9i9. Exons 8, 9 and 10 are indicated in red, blue and green, respectively. Primers used for RT-PCR are indicated as arrows. MO is indicated in red. Due to defective splicing, exon 9 is spliced out of the processed mRNA. De. RT-PCR showing *gapdh* control in Nacre (control) MO and Fer e9i9 MO injected embryos. RT-PCR showing *Fer* product in Nacre control MO and Fer e9i9 MO injected embryos with a decrease in size of the product in e9i9 MO injected embryos. Df. Sanger sequencing showing the exclusion of exon 9.

The identified peptide corresponded to tyrosine 714 (Y716 in zebrafish) which is the Fer autophosphorylation site, phosphorylation of which results in activation of Fer kinase activity [26]. Sequence alignment of the Fer kinase primary amino acid structure showed conservation of FER protein among vertebrates, from *Homo sapiens* to *Danio rerio* (Figure 2A), all coding for the conserved autophosphorylation site tyrosine. We cloned zebrafish *fer* from cDNA obtained from 1 dpf embryos. Sequence analysis confirmed that the *fer* transcript that was cloned was identical to ENSDART00000050957 or zebrafish *fer-201* encoding full-length Fer protein. Thus, Fer and its autophosphorylation site are evolutionarily conserved in vertebrates, including zebrafish.

As a first step to investigate the role of Fer kinase during development, *in situ* hybridization (ISH) was performed on zebrafish embryos, using a *fer*-specific probe (Figure 2B). As a negative control sense *fer* probe was used (Figure 2C). Ubiquitous expression of Fer is already apparent at the 8-cell stage, prior to zygotic transcription, indicating maternal contribution of *fer* mRNA. *Fer* remained ubiquitously expressed until 10 somite stage. At later stages, *fer* expression was enriched anteriorly and in the pectoral fins. In conclusion, *fer* expression was ubiquitous during gastrulation and more restricted during later stages of development.

To investigate the role of Fer *in vivo*, morpholinos against *fer* were injected into zebrafish embryos at the 1-cell stage. As a negative control, non-related Nacre MO was injected that does not induce developmental defects, as we have observed before [27]. We assessed Fer protein knockdown by immunoblotting using commercially available Fer antibodies, but unfortunately, these reagents did not allow detection of endogenous Fer in zebrafish embryos. Instead, knockdown of *fer* was verified by RT-PCR of knockdown embryos (Figure 2D). Injection of *fer* e5i6 MO blocked splicing of *fer* intron 5, resulting in the incorporation of intron 5 and introducing multiple stop codons in the aberrantly spliced *fer* mRNA (Figure 2Da and Db). Injection of *fer* e9i9 MO caused aberrant splicing, thereby excluding exon 9 of *fer* (Figure 2Dd and De). These effects of MO injections were verified by sequencing (Figure 2Dc and Df, respectively).

### Fer knockdown phenocopies Shp2 knockdown, NS and LS Shp2 expression in zebrafish

**Figure 3:**
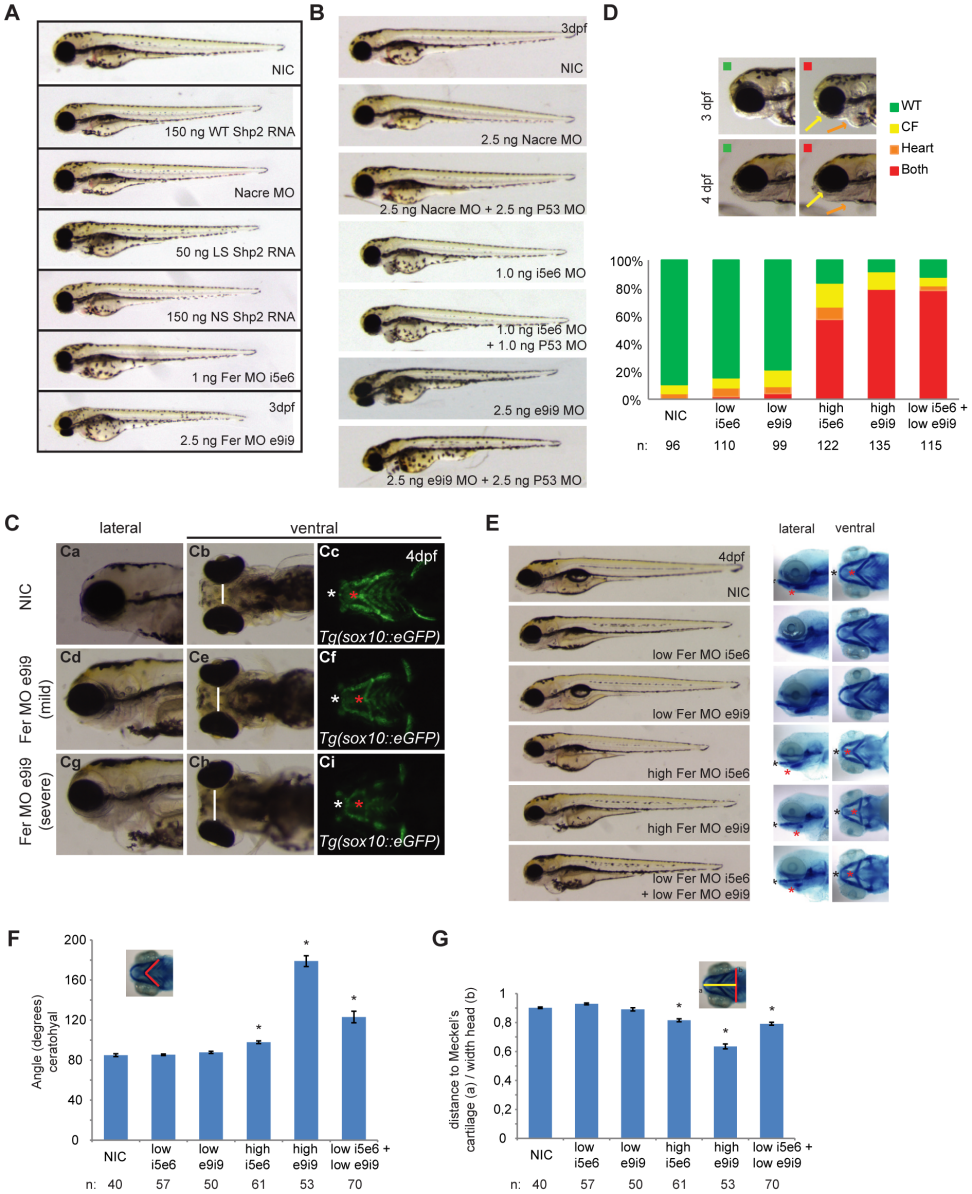
Fer knockdown induced craniofacial defects in zebrafish embryos. A. Embryos were injected at the 1-cell stage with 1 ng Fer i5e6 MO, 2.5 ng e9i9 MO or 2.5 ng Nacre MO as a negative control. Additionally, embryos were injected with 150 ng WT Shp2 RNA, 150 ng NS Shp2 RNA or 50 ng LS Shp2 RNA. B. Embryos were injected at the 1-cell stage with 2.5 ng Nacre control MO, 1.0 ng Fer i5e6 MO, 2.5 ng Fer e9i9 MO or in combination with 2.5 ng P53 MO. C. Craniofacial structures were imaged using *Tg(-4.9sox10:EGFP)^ba2^* embryos expressing eGFP in neural crest cells that also form the cartilage. Embryos were injected with Fer MO e9i9 at the 1 cell stage and imaged at 4dpf. Ceratohyal is indicated with a red asterisk and Meckel's cartilage with a white asterisk. Both moderate and severe phenotypes are depicted together with non-injected controls (NIC). D. Embryos were injected at the 1-cell stage with suboptimal concentrations of MO (0.5 ng i5e6 MO; *n* = 110 and 1.0 ng e9i9 MO; *n* = 99). High levels of both MO's (1.0 ng i5e6 MO; *n* = 122 and 2.5 e9i9 MO; *n* = 135) or low levels of both MO's were co-injected (*n* = 115). Embryos were imaged at 3 dpf and 4 dpf and grouped by having a WT appearance (green), a craniofacial defect alone (yellow) a heart defect alone (orange) or both (red) at 4 dpf. Relative levels of phenotypes are depicted. E. Embryos were injected at the 1-cell stage with normal dose of MO (1.0 ng i5e6 MO; *n* = 61 and 2.5 e9i9 MO; *n* = 53), low doses of MO (0.5 ng i5e6 MO; *n* = 57 and 1.0 ng e9i9 MO; *n* = 50) or a co-injected with low doses of MO (*n* = 70). Morphology at 4 dpf is depicted. Embryos were fixed and stained with Alcian blue at 4 dpf and imaged laterally and ventrally. For quantification, the angle of the ceratohyal (F) and the ratio of the distance from the back of the head to Meckel's cartilage and the width of the head was determined (G) (* indicates significance, Student's t-test p<0.005).

Injection of both Fer morpholinos (i5e6 and i9e9) induced developmental defects including reduced body axis length, heart edema and craniofacial defects at 3 dpf (Figure 3A). These defects were similar to the phenotypes observed in zebrafish expressing NS and LS Shp2 that were analyzed in parallel (Figure 3A). MOs are known to induce non-specific p53 activation and apoptosis [12]. To rule out that the observed phenotype is the mere result of p53 activation, co-injection of p53 MO was performed, which is an accepted control to assess specificity of MOs. Knockdown of p53 did not affect the *fer* knockdown phenotype, indicating that the phenotype was independent of p53 (Figure 3B). Taken together, two independent Fer MOs were used that blocked normal splicing of *fer* and induced developmental defects.

The craniofacial defects we observed in Fer knockdown embryos were reminiscent of the defects observed in Shp2 knockdown embryos as well as embryos expressing NS or LS Shp2 [9], [28]. To verify these craniofacial defects, we first used the *Tg(−4.9sox10:EGFP)^ba2^* transgenic line, which expresses GFP in neural crest-derived cartilage in the head [29]. We observed heart edema (Figure 3Ca, Cd and Cg) an increased distance between the eyes (Figure 3Cb, Ce and Ch), an increased angle of the ceratohyal (Figure 3Cc, Cf and Ci, red asterisk), a less protruded Meckel's cartilage (white asterisk), confirming the craniofacial defects. To verify the specificity of the Fer MOs again, we co-injected low doses of the two Fer-MOs that did not induce developmental defects on their own (Figure 3D). Co-injection of suboptimal doses of both Fer MOs together induced a drastic increase in the Fer knockdown phenotype with reduced body axis extension, heart edema (orange) and craniofacial defects (yellow) as prominent features (see Figure 3D lower panel). This was further verified by alcian blue stainings on Fer knockdown embryos at 4 dpf (Figure 3E). The angle of Meckel's cartilage (Figure 3F) and the ratio of the distance between the eyes and the back of the head to the ceratohyal (Figure 3G) were used to quantify these defects. Whereas NIC embryos showed an angle of 85±1 degrees (*n* = 40), injection of high levels of Fer MO e5i6 induced an increased angle of the ceratohyal (98±1 degrees, *n* = 61), as did injection of high levels of Fer MO e9i9 (angle = 179±5 degrees, *n* = 53) which were both significant (p<0.005 Student's t-test) (Figure 3F). The ratio of the distance from the back of the head to Meckel's cartilage and the width of the head at 4 dpf showed a similar significant increase (e5i6 MO: ratio = 0.82±0.01; e9i9 MO: ratio = 0.63±0.02, compared to NIC: ratio = 0.90±0.01; p<0.001, Student's t-test) (Figure 3G). Embryos injected with low doses of Fer MO e5i6 alone showed an angle of the ceratohyal of 85±1 degrees, *n* = 57 (n.s.) and a ratio of 0.93±0.01 (p<0.001). Low dose Fer MO e9i9 injected embryos showed an angle of 88±1, *n* = 50 (n.s.) and a ratio of 0.89±0.01 (n.s.). Co-injection of both MO's at low doses together resulted in a drastic increase of craniofacial defects. Quantification of the craniofacial defects of these embryos showed an angle of 123±6, *n* = 70 (p<0.001) and a ratio of 0.79±0.01 (p<0.001) (Figure 3E–G).

We tried to rescue the Fer MO-induced developmental defects by expression of mRNA encoding full length Fer. Unfortunately, despite many attempts, we failed to rescue the Fer MO induced developmental defects (Figure S2A). Expression of Fer by itself induced reduced body axis extension, cardiac edema and craniofacial defects (Figure S2B), which impaired assessment of the rescues. It is not uncommon that up- and down-regulation of signalling proteins, including Shp2 and RhoA, induce similar developmental defects [9]. In conclusion, knockdown of Fer was specific and resulted in craniofacial defects, heart edema and reduced body axis extension, which is reminiscent of the developmental defects in Shp2 knockdown embryos as well as NS and LS Shp2 expressing embryos.

### Fer knockdown induced convergence and extension defects

**Figure 4:**
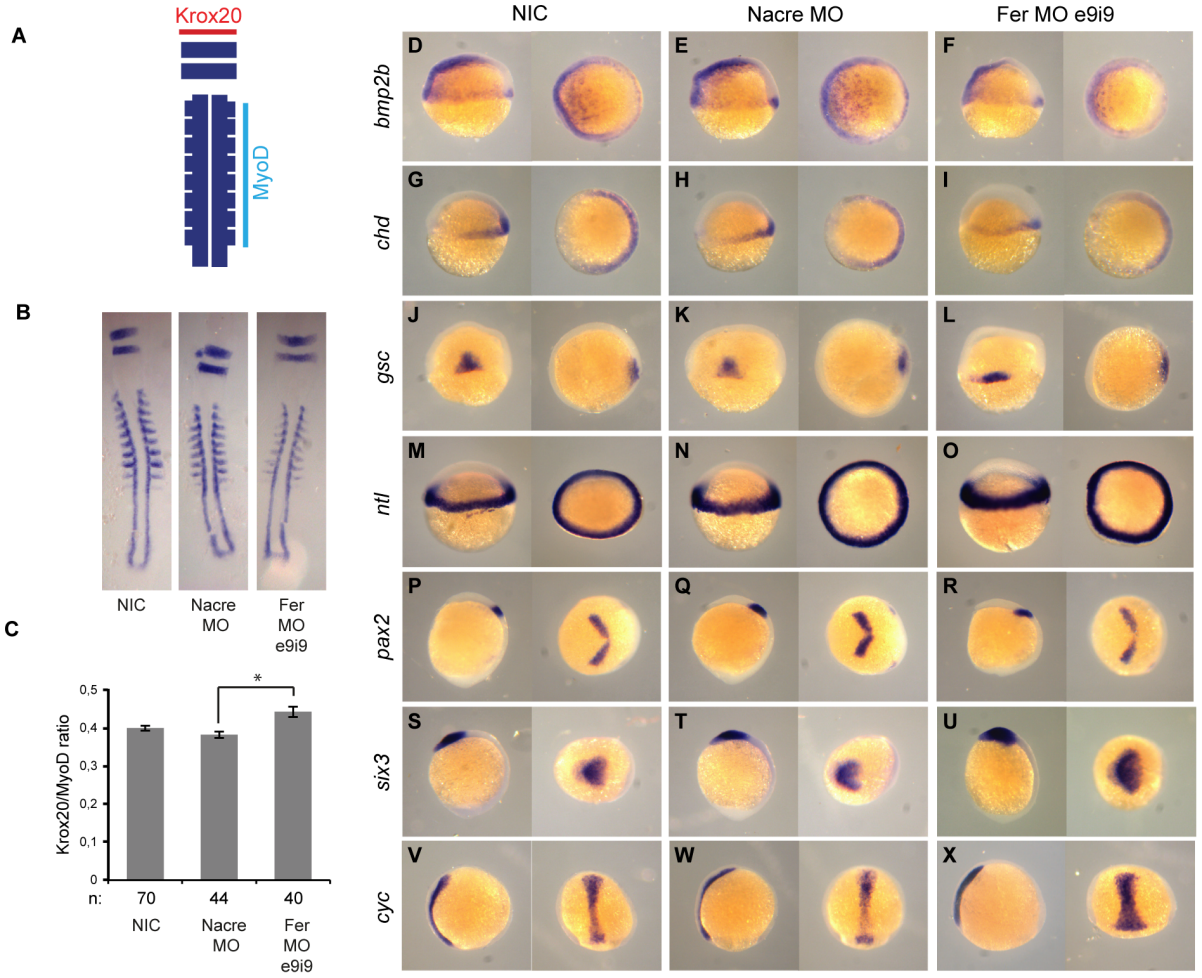
Fer knockdown resulted in C&E defects but not in changes in cell fate. A. *Krox20/myoD in situ* hybridization as a method to quantify C&E defects. *Krox20* (red) staining for rhombomeres 3 and 5 was used to measure the width, which correlates with convergence cell movements in the embryo. *MyoD* (light blue) staining for the somites was used to measure the length, *i.e.* extension of the embryo. The ratio of *Krox20/myoD* correlates directly with convergence & extension cell movements during gastrulation. B. Flatmounts of *krox20/myoD* stained NIC (*n* = 70), control MO (*n* = 44) and Fer MO (*n* = 40) embryos. C. The ratio of the width of a krox20-positive rhombomere and the length of 8 somites was determined (* indicates significance, Student's t-test p<0.005). D–X. Embryos were injected at the 1-cell stage with control MO or Fer e9i9 MO and subjected to ISH for various markers of cell fate determination. Note that the staining of the *gsc, pax2, six3* and *cyc* probes show broader and shorter expression in Fer knockdown embryos than in controls.

Fer knockdown phenocopied Shp2 knockdown and NS and LS expression at later developmental stages. Loss of Shp2 and expression of NS and LS variants of Shp2 induce C&E defects in developing zebrafish embryos [9]. We investigated whether Fer knockdown caused developmental defects during gastrulation as well. To investigate C&E defects upon Fer knockdown, embryos were subjected to *in situ* hybridization (ISH) using probes for *myoD*, staining the somites and *krox20* staining rhombomeres 3 and 5, a verified method to quantify C&E defects in developing zebrafish embryos (Figure 4A) [30], [31]. Indeed, Fer morphant embryos showed C&E defects compared to control morphants (Student's t-test, p<0.005) (Figure 4B and 4C). In addition to C&E defects, cell specification may be affected by the loss of Fer function. To investigate this, we subjected developing embryos to ISH using panel of cell fate markers, including *bmp2b*, *cyc*, *chd*, *ntl*, *six3*, *gsc* and *pax2* (Figure 4D–X). Expression levels of these markers were unchanged in Fer MO and control MO injected embryos compared to non-injected controls (NIC). However, expression of *gsc, pax2, six3* and *cyc* showed broader and shorter signal in Fer knockdown embryos, compared to NIC and control MO injected embryos, likely resulting from C&E defects (Figure 4 J–L, P–R, S–U and V–X, respectively). Thus, loss of Fer leads to C&E defects similar to Shp2 knockdown in developing zebrafish, and does not cause alterations in early cell fate determination.

### Fer knockdown cooperates with NS and LS, but not WT Shp2 expression

**Figure 5:**
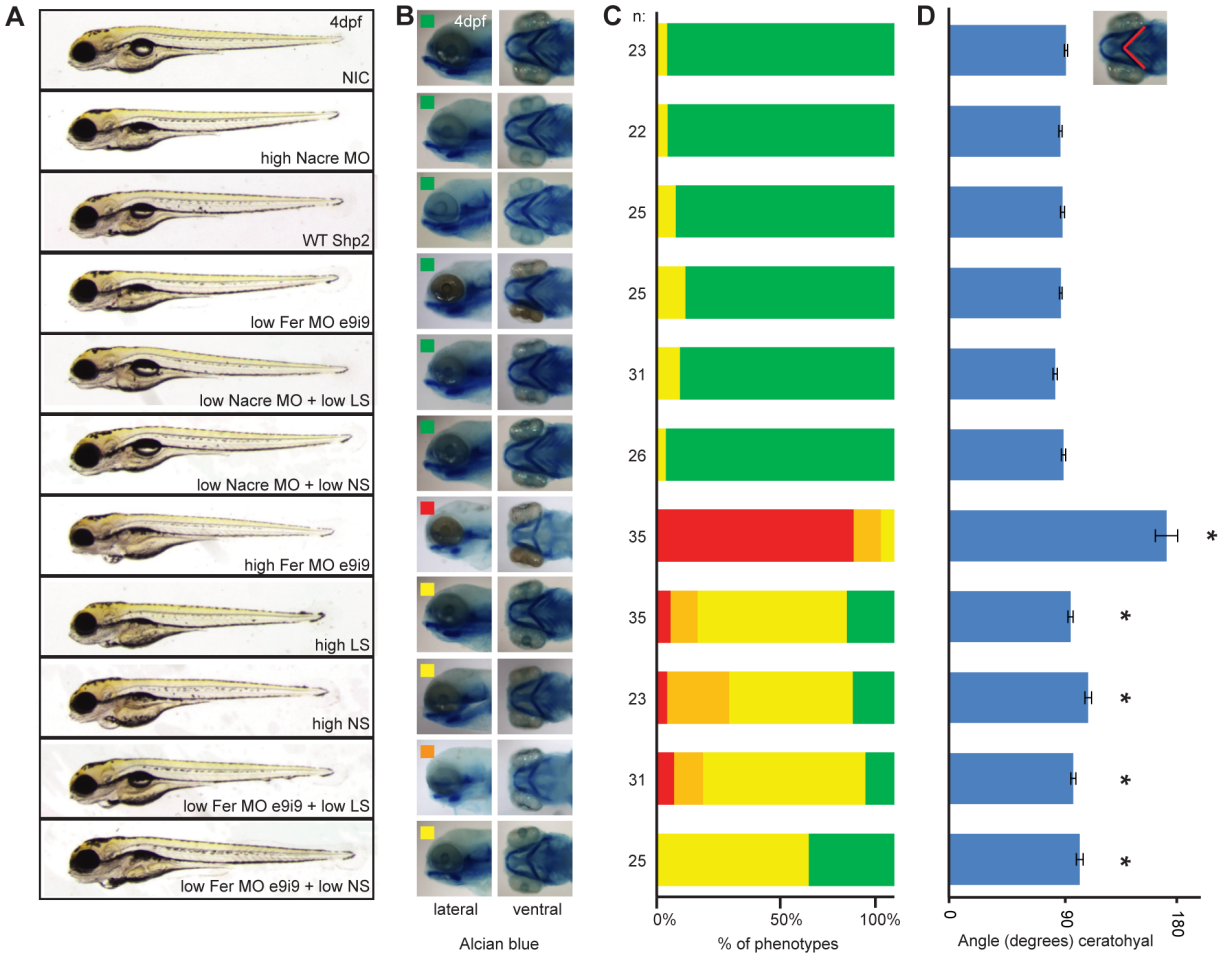
Partial knockdown of Fer cooperated with suboptimal expression of NS- and LS- but not WT Shp2 to induce developmental defects. A. Embryos were injected with control MO, Fer e9i9 MO and NS, LS or WT Shp2 mRNA as indicated, and imaged at 4 dpf. B. Alcian blue staining showing craniofacial defects scored on severity (green: wild type, yellow: mild phenotype, orange: moderate phenotype, red: severe phenotype). C. Embryos were scored based on craniofacial defect upon alcian blue staining. The number of embryos per condition is indicated. D. The angle of the ceratohyal was quantified as a measure of craniofacial defects (* p<0.005, Student's t-test).

Mass spectrometric analysis showed reduced Fer Y714 phosphopeptide. In developing zebrafish, we found that loss of Fer phenocopies Shp2 knockdown and expression of NS and LS Shp2, and leads to C&E defects. To investigate if downregulation of Fer contributes to the pathogenesis of NS and LS, we performed a genetic epistasis experiment, where we induced suboptimal knockdown of Fer in combination with injection of low amounts of NS and LS Shp2 mRNA that do not induce defects by themselves. Control injections only marginally affected craniofacial development. The ceratohyal angle in control injected embryos was 93±1 degrees, which was a slight increase compared to the ceratohyal angle of control injected embryos, 88±1 degrees (p<0.05). Whereas Fer knockdown caused developmental defects (Figure 5A, B), partial knockdown of Fer using a low dose of Fer MO e9i9 did not induce developmental defects (ceratohyal angle: 89±1 degrees, n.s). Likewise, injection of NS and LS, but not WT Shp2 mRNA at normal doses induced heart edema, reduced body axis extension and craniofacial defects (NS: ceratohyal angle: 110±3 degrees, p<0.005 and LS: angle: 96±2 degrees, p<0.001). At lower doses, NS and LS Shp2 also did not cause major developmental defects or an increase in the angle of the ceratohyal compared to control injections (Low NS: 91±2 degrees, n.s and low LS: 84±2 degrees, p<0.05). When combined with low doses of Fer MO however, NS and LS showed heart edema and major increases in craniofacial defects (Low NS + low Fer MO: 103±3 degrees, p<0.001 and Low LS + low Fer MO: 98±2 degrees, p<0.001) (Figure 5A, 5C and 5D). This indicates that a decrease in Fer cooperates with NS and LS to induce developmental defects seen in NS and LS.

## Discussion

Using a comparative phosphoproteomics approach focused on pTyr-containing proteins, we identified a phosphopeptide corresponding to Fer kinase as the main decreased phosphopeptide in zebrafish embryos expressing NS or LS mutant Shp2 compared to WT. Fer knockdown induced developmental defects in zebrafish embryos that are reminiscent of defects induced by NS and LS Shp2. Epistatic interaction analyses suggested a genetic interaction between Fer kinase and mutant Shp2.

Previously, we used comparative phosphoproteomics in zebrafish to identify differences between wild type and Fyn/Yes knockdown embryos [18]. At that time, TiO2 columns were used to enrich for phosphopeptides, and only highly abundant phosphoserine- and phosphothreonine-containing phosphopeptides were identified, excluding low abundant pTyr-containing peptides [18]. Improved methods using immunoprecipitation of pTyr peptides allow for the identification of endogenous tyrosine phosphorylated peptides [27]. Stable isotope dimethyl labelling allows for the quantitative comparison of three different samples in an economical fashion, compared to other peptide labelling methods [17]. Other groups have successfully analysed the proteome of zebrafish under different conditions [32]–[34], however these experiments often focussed on the whole proteome in a specific organ. Given the crucial role of tyrosine phosphorylation in signalling during development, we hypothesized that specific analysis of the tyrosyl phosphoproteome would provide insights into development and disease. Particularly in case of NS and LS, two syndromes that are associated with expression of mutant Shp2, a protein-tyrosine phosphatase. Recently, a MS based approach was used to identify proteins that were affected by the altered binding properties of NS and leukemia associated Shp2 [35]. Although highly informative, these experiments were performed using the tandem SH2 of Shp2 expressed in cells. As full length NS and LS Shp2 exhibit different dynamic properties than WT [36], [37], using the full length protein under physiological conditions *in vivo* gives more relevant insights into the etiology of the disease. Therefore, we performed for the first time a comparative phosphoproteomics experiment in zebrafish using pTyr immunoprecipitation and stable isotope dimethyl labelling.

Using this technique, we identified several phosphopeptides that were increased or decreased in NS and LS Shp2 embryos. One of these peptides corresponded to the Fer autophosphorylation site and was the most strongly decreased phosphopeptide in the zebrafish disease model for NS and LS. Follow-up experiments showed that Fer mRNA is maternally contributed and is expressed ubiquitously during gastrulation. Later, Fer expression is restricted anteriorly and in the pectoral fins of developing zebrafish embryos (Figure 2B). Fer is conserved and *fps/fes/fer* homologues exist in sponges (Fes/FER_SR), *D. melanogaster* (dfps85D) and *C. elegans* (FRK-1) [38], [39]. In chick, Fer expression is ubiquitous, with higher expression levels during development than at later stages [24]. Also in mammalian cells, Fer expression is widely distributed [23]. Fer expression is vital for dorsal closure of *C.elegans*, yet its kinase activity seems to be redundant in development [39]–[41]. We demonstrate that in fish, a decrease in total Fer levels by MO-induced knockdown resulted in developmental defects that were reminiscent of Shp2 knockdown, and NS and LS expression in zebrafish (Figure 3A) [9]. Moreover, loss of Fer resulted in C&E defects during gastrulation, which were also observed in NS and LS zebrafish (Figure 4) [9], [31]. Indeed, also at later stages craniofacial malformations and heart edema were observed in Fer knockdown embryos, similar to NS and LS Shp2 expressing embryos (Figure 3) [9], [28], [31], [42]. We show a genetic interaction between *fer* and *ptpn11*, since loss of Fer contributes to the NS and LS phenotype (Figure 5).

The mechanism underlying the role of Fer kinase in development remains to be determined definitively. Fer has been reported to be involved in various signaling pathways. Expression of full length *fer* is essential for *Drosophila* gastrulation via Src42A, a Drosophila Src homologue [40] However, it is unclear if kinase activity is essential for this function of Fer. In *C.elegans*, FRK-1 is essential for embryonic closure and morphogenesis but this is not dependent on FRK-1 kinase activity [39]. Depletion of Fer kinase activity leads to a reduction in p38 MAPK but not ERK activity in activated mast cells [43] Fer is able to sustain ERK activation under hypoxic conditions but kinase activity is not essential [44]. Interestingly, Fer kinase activity is essential for β-catenin-cadherin complex formation. Expression of kinase dead Fer attenuates formation of this complex. EGF treatment rescues this defect, indicating a compensatory mechanism for the loss of Fer kinase activity. Interestingly, PTP1B, another member of the PTP family, associates with Cadherin in a Fer kinase-dependent manner, but whether PTP1B dephosphorylates Fer remains to be determined [45]. How NS and LS Shp2 cause a downregulation of Fer protein tyrosine phosphorylation remains to be determined. Since both activated NS Shp2 and catalytically impaired LS Shp2 have similar effects on Fer, it is unlikely that Fer is a direct substrate of Shp2. Disease associated Shp2 was found to bind to hyperphosphorylated substrates, both in NS and LS and in experimental conditions [20], [46]. Fer and Shp2 share many interacting partners, including PDGFR, integrins, PECAM-1 and others [25], [46]–[48]. In addition, Fer and Src (an important Shp2 interacting protein) share many substrates, including cortactin [41], [49]. Disease associated Shp2 exhibits altered dynamics and binding properties [35]–[37]. Mutant Shp2 may quench binding proteins that are normally required for full activation of Fer kinase, resulting in reduced tyrosine phosphorylation of Fer upon expression of NS and LS Shp2.

Alternatively, Fer activity might be downregulated through enhanced growth factor signaling that occurs in NS and LS [36], [45]. While many studies have shown a loss of phosphatase activity in LS Shp2, the ability of LS Shp2 to enhance ERK activation remains subject of debate [4], [36]. In zebrafish, we observe enhanced ERK activation in both NS and LS [50] and hence, reduced Fer phosphorylation may result from a negative feedback mechanism, resulting from enhanced growth factor signaling. In conclusion, we identify Fer kinase as a likely downstream target of NS and LS Shp2 and we provide evidence that deregulation of Fer enhanced the NS and LS phenotype *in vivo*.

## Supporting Information

Figure S1 **MS2 spectrum of the identified Fer peptide.** Fer peptide sequence is shown in the upper left corner indicating the y- and b-ions. Annotated ions are indicated with their respective m/z values.
(TIF)

Figure S2 **Attempted rescue of Fer MO and overexpression of Fer.** Embryos were injected at the 1-cell stage with 2.5 ng Fer MO e9i9 either alone or in combination with increasing amounts of synthetic Fer mRNA (5 ng, 25 ng, 37.5 ng, 50 ng, 100 ng, 150 ng, 200 ng, 250 ng). Co-injection of Fer mRNA did not rescue the Fer knockdown phenotype. B. embryos were injected at the 1-cell stage with Fer WT mRNA. Injection of Fer mRNA by itself induced craniofacial defects, shorter length and heart edema.
(TIF)

Table S1 **Raw MS data.** Tab1 “PD_Output” shows raw, non-normalized data from Proteome Discoverer, which is used for normalization on all nonphosphopeptides. “numberOFphospho”: number of phosphoresidues present in the peptide. “numberOFlocalized”: number of localized phosphoresidue (pRS>75). “peptideSite”: localized site. Tab2 “Aggregation” shows data averaged per rows based on the following values (protein group accession, sequences, description, numberofphospho, numer of localized, peptide sites). “SITES” tab shows all normalized, quantified, localized, protein assigned peptides. “SITES with lacking protein” tab shows all normalized, quantified, localized, peptides, including non-protein assigned peptides. “quantified only pRS<75” shows all normalized quantified peptides for which no phosphosite could be assigned. “all quant phosphopepts combined” shows a combination of “SITES with lacking protein” and “quantified only pRS<75” with duplicate peptides for which one site is assigned and the other is not removed. “Table 1” shows “all quant phosphopepts combined” where non-assigned peptides are BLASTed against the zebrafish proteome to identify the protein, phosporylation site in zebrafish and the phosphosite is compared to human homologs from PhosphoSite.org to identify the site. Sites without a human homolog were removed from the analysis.
(XLSX)
