# Supplementary figures and images for: Phosphoproteomics-Mediated Identification of Fer Kinase as a Target of Mutant Shp2 in Noonan and LEOPARD Syndrome
Source: PLoS One. 2014 Sep 3;9(9):e106682. doi: 10.1371/journal.pone.0106682 (PMC4153654; doi:10.1371/journal.pone.0106682)

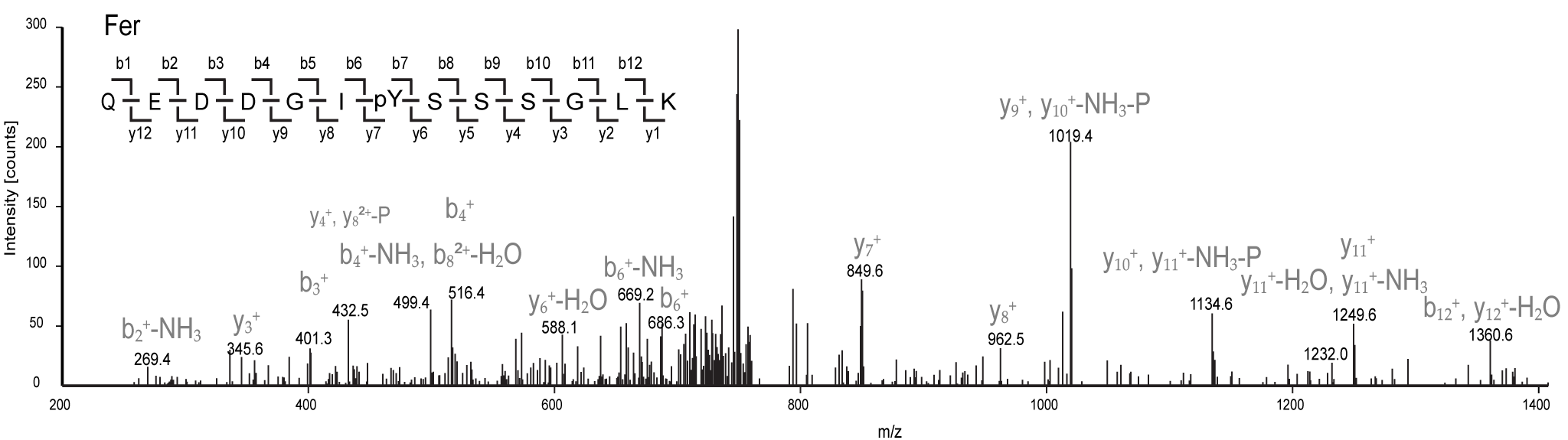

Supplement: Figure S1 — MS2 spectrum of the identified Fer peptide. Fer peptide sequence is shown in the upper left corner indicating the y- and b-ions. Annotated ions are indicated with their respective m/z values. (TIF) [file pone.0106682.s001.tif]

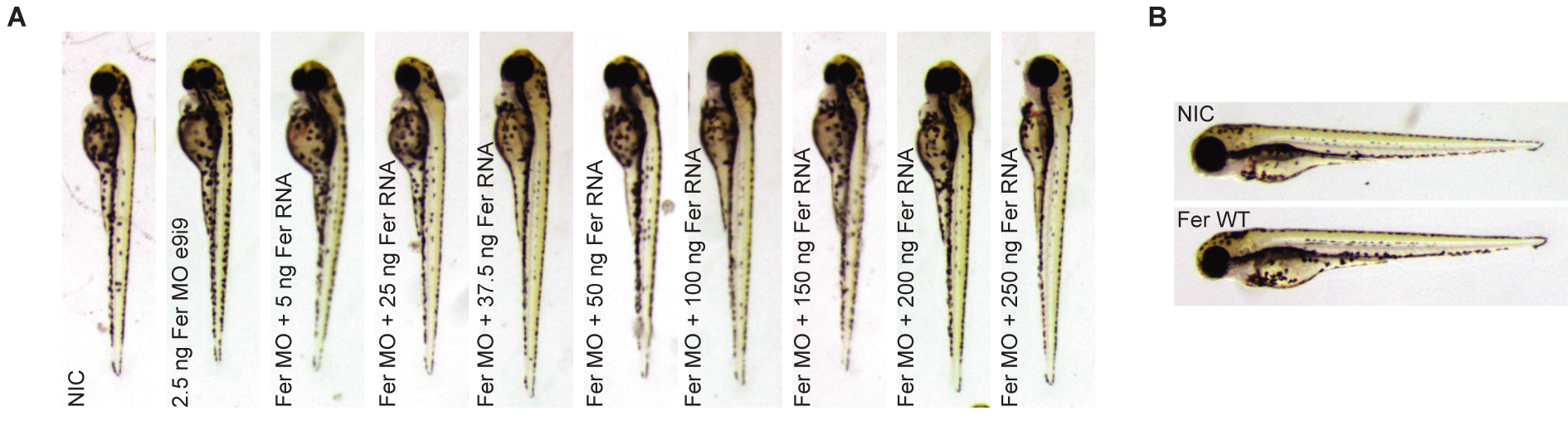

Supplement: Figure S2 — Attempted rescue of Fer MO and overexpression of Fer. Embryos were injected at the 1-cell stage with 2.5 ng Fer MO e9i9 either alone or in combination with increasing amounts of synthetic Fer mRNA (5 ng, 25 ng, 37.5 ng, 50 ng, 100 ng, 150 ng, 200 ng, 250 ng). Co-injection of Fer mRNA did not rescue the Fer knockdown phenotype. B. embryos were injected at the 1-cell stage with Fer WT mRNA. Injection of Fer mRNA by itself induced craniofacial defects, shorter length and heart edema. (TIF) [file pone.0106682.s002.tif]
